# Ionic homeostasis in brain conditioning

**DOI:** 10.3389/fnins.2015.00277

**Published:** 2015-08-10

**Authors:** Ornella Cuomo, Antonio Vinciguerra, Pierpaolo Cerullo, Serenella Anzilotti, Paola Brancaccio, Leonilda Bilo, Antonella Scorziello, Pasquale Molinaro, Gianfranco Di Renzo, Giuseppe Pignataro

**Affiliations:** ^1^Division of Pharmacology, Department of Neuroscience, Reproductive and Dentistry Sciences, School of Medicine, Federico II University of NaplesNaples, Italy; ^2^SDN Istituto di Ricerca Diagnostica e NucleareNaples, Italy; ^3^Division of Neurology, Department of Neuroscience, Reproductive and Dentistry Sciences, School of Medicine, Federico II University of NaplesNaples, Italy

**Keywords:** transporters, ionic exchangers, stroke, preconditioning/post-conditioning, brain ischemia

## Abstract

Most of the current focus on developing neuroprotective therapies is aimed at preventing neuronal death. However, these approaches have not been successful despite many years of clinical trials mainly because the numerous side effects observed in humans and absent in animals used at preclinical level. Recently, the research in this field aims to overcome this problem by developing strategies which induce, mimic, or boost endogenous protective responses and thus do not interfere with physiological neurotransmission. Preconditioning is a protective strategy in which a subliminal stimulus is applied before a subsequent harmful stimulus, thus inducing a state of tolerance in which the injury inflicted by the challenge is mitigated. Tolerance may be observed in ischemia, seizure, and infection. Since it requires protein synthesis, it confers delayed and temporary neuroprotection, taking hours to develop, with a pick at 1–3 days. A new promising approach for neuroprotection derives from post-conditioning, in which neuroprotection is achieved by a modified reperfusion subsequent to a prolonged ischemic episode. Many pathways have been proposed as plausible mechanisms to explain the neuroprotection offered by preconditioning and post-conditioning. Although the mechanisms through which these two endogenous protective strategies exert their effects are not yet fully understood, recent evidence highlights that the maintenance of ionic homeostasis plays a key role in propagating these neuroprotective phenomena. The present article will review the role of protein transporters and ionic channels involved in the control of ionic homeostasis in the neuroprotective effect of ischemic preconditioning and post-conditioning in adult brain, with particular regards to the Na^+^/Ca2^+^ exchangers (NCX), the plasma membrane Ca2^+^-ATPase (PMCA), the Na^+^/H^+^ exchange (NHE), the Na^+^/K^+^/2Cl^−^ cotransport (NKCC) and the acid-sensing cation channels (ASIC). Ischemic stroke is the third leading cause of death and disability. Up until now, all clinical trials testing potential stroke neuroprotectants failed. For this reason attention of researchers has been focusing on the identification of brain endogenous neuroprotective mechanisms activated after cerebral ischemia. In this context, ischemic preconditioning and ischemic post-conditioning represent two neuroprotecive strategies to investigate in order to identify new molecular target to reduce the ischemic damage.

## Ischemic preconditioning

The concept of ischemic preconditioning was introduced first in the heart (Murry et al., 1986) and later on in the brain (Murry et al., 1986; Schurr et al., 1986). Ischemic preconditioning is neuroprotective strategy in which a subliminal stimulus is applied before a subsequent harmful ischemia (Kirino, 2002; Dirnagl et al., 2003; Gidday, 2006). The state of tolerance mediated by this strategy is composed of two phases of neuroprotection. The first phase, named *rapid or acute preconditioning* (Perez-Pinzon et al., 1997), starts 3–5 min after the preconditioning stimulus and ends 1 h later and is due to rapid post-translational protein modifications (Barone et al., 1998; Meller et al., 2006). The second phase, named *delayed preconditioning* (Perez-Pinzon et al., 1997), starts 2–3 days after preconditioning and ends 1 week later and mainly involves *de novo* protein synthesis (Barone et al., 1998; Dirnagl et al., 2003; Meller et al., 2005).

### Mechanisms involved in ischemic preconditioning neuroprotection

The molecular mechanisms underlying ischemic tolerance are not yet fully understood because of its extreme complexity, involving many signaling pathways and alterations in gene expression. Several studies have investigated the signaling cascades and key molecules both in *in vitro* and *in vivo* models of ischemic preconditioning. Most of the stressors induce both rapid and delayed tolerance. As recently reviewed (Obrenovitch, 2008), the features of ischemic tolerance partially are similar to naturally occurring adaptive mechanisms, including at cellular levels:

1. Alterations in cellular energy metabolism: preconditioning preserves mitochondrial function (Dave et al., 2001; Racay et al., 2007) and mediates an increase in expression of genes playing a role in energy metabolism.
2. Preservation of mitochondrial membrane potential: preconditioning preserves respiratory complexes, mitochondrial oxidative phosphorylation and antioxidant enzymes such as superoxide dismutases (SODs), catalase, glutathione peroxidase, and thioredoxin system (Danielisová et al., 2006; Racay et al., 2007).
3. Modulation of neuronal excitotoxicity: preconditioning mediates a shift to inhibitory neurotransmission, suppressing glutamate release, downregulating AMPA and NMDA receptors, and glial glutamate transporters, increasing GABA release and GABA-A receptors expression (Dave et al., 2005).
4. Suppression of cell death and apoptotic mechanisms: preconditioning reduces the release of cytochrome c, inhibits caspases, and proapoptotic genes, and activates survival pathways such as serine/threonine activated kinases (Akt) and extracellular signal-regulated kinases (ERK) and trophic factors (Lehotský et al., 2009).
5. Activation of the mechanisms mediating DNA repair and plasticity by increasing the activity nerve growth factor (NGF) and brain-derived neurotrophic factor (BDNF) (Gidday, 2006; Lehotský et al., 2009).
6. Reduction of inflammatory response: preconditioning activates Toll-like receptor, suppresses induced proinflammatory cytokines (TNFs) and activates transcription nuclear factor κB (NFκB) (Gidday, 2006).
7. Cerebrovascular adaptation by vascular remodeling: preconditioning activates vascular endothelial growth factors (VEGF), erythropoietin and heme oxygenase 1 as targets of hypoxia inducing factor 1α (HIF-1α). The blood–brain barrier was preserved thanks to the reduced activity of matrix metalloproteinases and cell adhesion molecules (Gidday, 2006).
8. Improved capacity to preserve cellular ionic and pH homeostasis: preconditioning regulates expression and activity of ion transport systems (Ca2^+^-ATPase, acid-sensing ion channels, Na^+^/H^+^ exchange, Na^+^/K^+^/2Cl^−^ cotransport and Na^+^/Ca2^+^ exchanger) and transporters for metabolites.

## Ischemic post-conditioning

Unlike ischemic preconditioning, ischemic post-conditioning is a relatively novel neuroprotective strategy (Zhao et al., 2006; Pignataro et al., 2008). Rapid revascularization of the occluded vessels represent one of the most effective approaches currently used for the treatment of acute ischemic stroke. However, during the early reperfusion phase an increase in reactive oxygen species and intracellular free Ca^2+^ may occur, thus leading to additional injury (Kuroda and Siesjö, 1997). Ischemic post-conditioning is realized by applying brief interruptions of reperfusion after ischemia. That a protocol of cycles of brief reperfusion followed by re-occlusion was able to reduce the ischemic damage was first demonstrated during cardiac ischemia (Zhao et al., 2003). More recently, it has been demonstrated that ischemic post-conditioning induces neuroprotection also in a rat hippocampal slice model of cerebral ischemia (Scartabelli et al., 2008), in rodent models of spinal cord injury (Jiang et al., 2006), and in models of focal (Zhao et al., 2006; Pignataro et al., 2008, 2013b; Xing et al., 2008), and global ischemia (Pateliya et al., 2008; Wang et al., 2008).

### Mechanisms involved in ischemic post-conditioning neuroprotection

**Figure 1:**
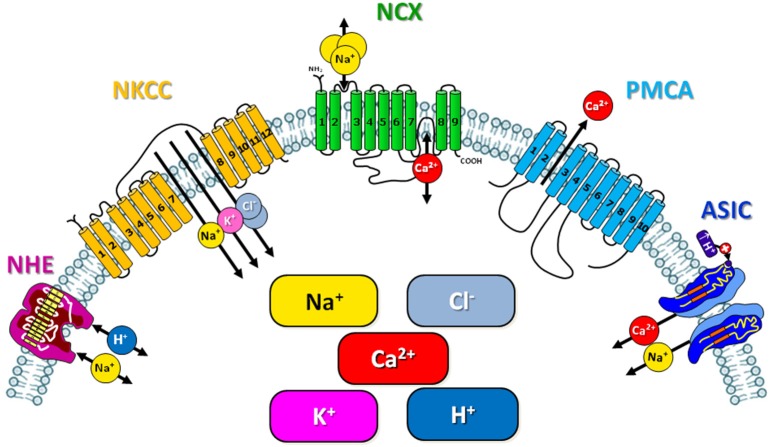
**Schematic representation of the ionic exchangers/transporters involved in the neuroprotection mediated by ischemic preconditioning and post-conditioning**.

At present a few information are available about the mechanisms involved in the neuroprotection mediated by ischemic post-conditioning. Since post-conditioning is applied after the ischemic injury, the changes observed in the brain have to be ascribed to the combined effects of post-conditioning and stroke, rather than the effect of the post-conditioning only. Thus, it is difficult to sunder the protective mechanisms of post-conditioning from the consequences of its protection. However, up until now, many mechanisms have been suggested (Zhao, 2009), such as:

1. Reduction of hyperemia consequent to the reperfusion, that may be accompanied other toxic events, such as the production of free radicals, loss of blood–brain barrier integrity, and activation of inflammation cascade (Schaller and Graf, 2004).
2. Blocking of apoptosis. Ischemic post-conditioning reduced ROS production (Zhao et al., 2006), attenuates lipid peroxidate levels (Xing et al., 2008) and increased the activities of antioxidant enzymes, such as superoxide dismutase and catalase (Danielisová et al., 2006). Furthermore, rapid post-conditioning reduced the release of cytochrome c from the mitochondria to the cytosol, a critical cascade for the induction of apoptosis, thus suggesting that post-conditioning may reduce ischemic injury by blocking apoptosis (Zhao et al., 2012).
3. Inhibition of inflammation. Rapid post-conditioning inhibits inflammation after stroke. Indeed, rapid post-conditioning inhibits myeloperoxidase activity, an indicator of leukocyte accumulation (Xing et al., 2008), attenuates the expressions of IL-1β, TNF-α, and the ICAM-1 protein expression (Xing et al., 2008).
4. Activation of Akt Pathway. In agreement with previous studies demonstrating that the Akt pathway promotes neuronal survival after stroke (Zhao et al., 2006), rapid post-conditioning increases Akt phosphorylation and Akt activity (Gao et al., 2008; Pignataro et al., 2008). Furthermore, the protection mediated by rapid post-conditioning was prevented by Akt inhibition (Gao et al., 2008; Pignataro et al., 2008) and abolished the protective effect post-conditioning in hippocampal slice cultures (Scartabelli et al., 2008), overall, ischemic post-conditioning exerts its neuroprotective effectiveness also through Akt activation.
5. Cellular ionic homeostasis and energy metabolism. Post-conditioning-mediated neuroprotection is correlated to the maintainance of ionic homeostasis occurring by modulating expression and activity of several proteins involved in ionic homeostasis, some of which will be treated below (Figure 1, Table 1).

**Table 1:** **Role of ionic exchangers/transporters during pre-and post-conditioning**.

**Ionic exchanger/Transporter**	**Role during preconditioning**	**Role during post-conditioning**	**References**
Na^+^/K^+^/2CI^−^ Cotransport (NK CC)	**Neuroprotective role:** Activation of NKCC participates in the protection mediated by ischemic cardiac preconditioning	**Neuroprotective role:** Activation of NKCC1 mimiks an *in vitro* post-conditioning	Schaefer et al., 1995; Yang et al., 2013
Na^+^/H^+^ Exchange (NHE)	**Neurodegenerative role:** NHE inhibition delays pH recovery thus mediating protection	**Neurodegenerative role:** NHE inhibition delays pH recovery thus mediating protection	Shipolini et al., 1997; Goldberg et al., 2002
ASIC	**Neurodegenerative role:** ASIC expression decrease during conditioning	**Neurodegenerative role:** ASIC expression decrease during conditioning	Pignataro et al., 2011a
NCX	**Neuroprotective role:** NCX expression increase during conditioning and its inhibition or silencing prevents neuroprotection	**Neuroprotective role:** NCX expression increase during conditioning and its inhibition or silencing prevents neuroprotection	Pignataro et al., 2011b, 2012
PMCA	**Neuroprotective role:** PM CA 1 is increased in the hippocampus following preconditioning	Role not known	Shimazaki et al., 1998

## Brain ionic transporters during ischemic conditioning

Ischemic brain conditioning activates intracellular biological responses to a potential lethal insult; therefore it is predictable that organs reinforce their tolerance when exposed to a sublethal insult prior or after a harmful episode of ischemia by increasing energy metabolism or delaying anoxic depolarization after the onset of the subsequent ischemic insult (Stenzel-Poore et al., 2003; Pignataro et al., 2008, 2009). Indeed, an impairment in the activity of voltage-gated potassium channels has been observed in cortical neurons exposed to a brief non-injurious oxygen and glucose deprivation (OGD). Moreover, it has been demonstrated that ischemic conditioning prevented the inhibition of Na^+^/K^+^-ATPase activity after brain ischemia in hippocampal and cortical neurons of rats subjected to global forebrain ischemia. Instead, *in vivo* experiments in gerbils showed that both Ca^2+^-ATPase activity and mitochondrial calcium internalization increased in CA1 hippocampal neurons exposed to preconditioning (Ohta et al., 1996). Accordingly, in hippocampal neurons obtained from preconditioned gerbils [Ca^2+^]_i_ increased after anoxic and aglycemic episodes whereas this increase was inhibited in ischemic-tolerant animals (Shimazaki et al., 1998). The molecular mechanisms of this phenomenon are still under investigation. An explanation may be the increase in the expression of the plasma membrane calcium ATPase 1 (PMCA-1) as recently demonstrated (Kato et al., 2005). Moreover, many evidence support the idea that other transporters are involved in the protection mediated by brain conditioning. In this regard, a key role is exerted by the sodium calcium exchanger (NCX), in fact its expression was reduced during cerebral ischemia (Pignataro et al., 2004; Boscia et al., 2006). More important, NCX downregulation was prevented when rats were subjected either to ischemic preconditioning or post-conditioning (Pignataro et al., 2013a). In addition Na^+^/H^+^ exchanger and Na^+^/K^+^/2Cl^−^ cotransporter are critical ion transporters in the context of cerebral ischemia, as they contribute to the regulation of intracellular pH and cell volume (Pedersen, 2006). Finally, acid-sensing cation channels (ASIC) have also been studied during ischemic conditions since they represent key targets in many different aspects of acidosis, a condition that occurs during ischemic stroke. Brain preconditioning as an adaptive response to stroke requires in particular new protein transcription and translation; giving that HIF-1–dependent transcription is being clearly common in activating pathways of several exchangers/transporters/ionic channels involved in stroke, scientific community has assigned a key role to HIF-1α, as a master transcriptional regulator of cellular and developmental response to hypoxia. In effect stable HIF-1 can bind to its heterodimeric partner HIF-1β, and together these proteins, once stabilized during hypoxic-ischemic conditions, can act in the nucleus to transactivate genes involved in adaptation to hypoxic-ischemic stress. HIF represents the “hyphen” that definitely binds to each other all the different mechanisms converging on the same signaling cascade, responsible for the homeostatic regulation in protected brain (Ratan et al., 2004). In the light of these premises, the present article will review the main role of protein transporters and exchangers controlling ionic homeostasis in the neuroprotective effect of ischemic preconditioning and post-conditioning, with particular regards to the Na^+^/Ca^2+^ exchangers (NCX), the plasma membrane Ca^2+^-ATPase (PMCA), the Na^+^/H^+^ exchange (NHE), the Na^+^/K^+^/2Cl^−^ cotransport (NKCC) and the acid-sensing cation channels (ASIC).

### Role of Na^+^/H^+^ exchange (NHE) and Na^+^/K^+^/2Cl^−^ cotransport (NKCC) in brain conditioning

The ubiquitous plasma membrane Na^+^/H^+^ exchanger is protein highly conserved across vertebrate species and characterized as a major membrane transporter involved in the regulation of cellular pH and volume in the nephron of the kidney. Specifically it is present in the intercalary cells of the collecting duct (Pedersen, 2006). The Na^+^/K^+^/2Cl^−^ cotransporter (NKCC) is a protein that aids in the active transport of sodium, potassium, and chloride into and out of cells; it is widely distributed throughout the body and it has important functions in organs that secrete fluids. NKCC2 is found specifically in the kidney, where it serves to extract sodium, potassium, and chloride from the urine so that they can be reabsorbed into the blood (Haas, 1994).

Na^+^/H^+^ exchanger and Na^+^/K^+^/2Cl^−^ cotransporter are critical ion transporters in the context of cerebral ischemia, as they contribute to the regulation of intracellular pH and cell volume (Pedersen, 2006). In particular, while the mammalian NHE is an integral membrane transport protein that mediates an electroneutral 1:1 exchange of intracellular H^+^ for extracellular Na^+^ (Xia et al., 2003), the electroneutral NKCC contransporter mediates the influx of Na^+^, K^+^, and Cl^−^ with a stoichiometry of 1Na^+^:1K^+^:2Cl^−^.

Due to this dual role it is difficult to predict whether a change in the expression and in the activity of these transporters may ameliorate or impair the susceptibility of cells to ischemia; for example, enhanced Na^+^/H^+^ exchange would help to reduce the intracellular pH but, on the other hand, it would promote cell swelling and further Ca^2+^ influx through Na^+^/Ca^2+^ exchange. In particular, in cortical astrocyte cultures obtained from Na^+^/H^+^ exchanger isoform 1-deficient NHE1(-/-) mice the lack of NHE1 attenuated the damage induced by conditions mimicking cerebral ischemia *in vitro*, and this effect was confirmed also *in vivo* (Kintner et al., 2004). Indeed, the pharmacological blockade of NHE-1 reduces infarct volume in animal models of brain ischemia. In addition, ischemia/reperfusion causes an increase in NHE-1 expression/activity (Ramasamy et al., 1995).

Although NHE activation is essential for the restoration of physiological pH_i_, hyperactivation of NHE1 in neurons, in response to the metabolic acidification associated with an ischemic–hypoxic insult (Vornov et al., 1996; Luo et al., 2005; Hwang et al., 2008; Kersh et al., 2009), disrupts the intracellular ion balance, causing intracellular Na^+^ and Ca2^+^ overload (Matsumoto et al., 2004), which eventually leads to cell death. Consequently the *in vitro* and *in vivo* approach of several studies have demonstrated neuroprotection with NHE inhibitors.

Similar data were obtained with genetic manipulations of NKCC1 in mice and by the inhibition of NKCC1 using bumetanide (Chen and Sun, 2005). NKCC1 is expressed in neurons throughout the brain where it contributes to the maintenance of [Cl^−^]_i_. Thus, it may affect neuronal excitability by regulating [Cl^−^]_i_. Expression of NKCC1 protein has also been found in astrocytes and oligodendrocytes (Lenart et al., 2004). The pharmacological inhibition or transgenic ablation of NKCC1 significantly attenuates infarction and swelling after brain ischemia induced by transient middle cerebral artery occlusion (tMCAO) (Chen and Sun, 2005). Therefore, it may be hypothesized that NKCC1 represents an attractive target for conditioning strategies against stroke.

### NHE and NKCC role during ischemic preconditioning

NHE and NKCC role during ischemic preconditioning is still controversial and very little is known about their role in the processes triggered by brain preconditioning, since all the studies were conducted in the heart. According to a first theory, activation of both transporters participates in the protection mediated by ischemic cardiac preconditioning. Indeed, the reduced acidification occurring during ischemia (Schaefer et al., 1995) along with the increase in intracellular sodium following preconditioning is consistent with a modulation of these proteins by preconditioning (Ramasamy et al., 1995). These mechanisms may include the activation of both NKCC and NHE, potentially resulting in reduced intracellular acidosis during ischemia. NKCC regulation is multifactorial, with evidence that the cotransporter is regulated by changes in cell volume (O'Donnell, 1993) and cAMP-dependent (Pewitt et al., 1990; Lytle et al., 1992; Klein, 1993) and non cAMP-dependent protein phosphorylation as well as ionic concentration gradients. Increased inward cotransporter flux during ischemia after preconditioning could be beneficial in protecting the heart through its functional coupling with the Cl^−^/HCO3^−^ exchanger (Anderson et al., 1990). In fact, chloride transported into the cell by the Na^+^-K^+^-2Cl^−^ cotransporter may exit via Cl^−^/HCO^−^_3_ exchange, thus increasing intracellular HCO3^−^ and limiting acidosis.

The primary factors regulating NHE include the intracellular proton concentration and the phosphorylation state. Under baseline conditions, the exchanger contributes to the extracellular/intracellular proton gradient with net outward transport of protons (Piwnica-Worms et al., 1986). Under conditions of ischemia in which protons are generated and intracellular pH decreases, the exchanger is stimulated to increase extrusion of protons, thus increasing the inward transport of sodium ions. However, this hypothesis has been more recently disproved. In fact, it has been highlighted that the above discussed study was carried out in hearts perfused with a medium containing HCO3^−^ and the method used to induce acute intracellular acidosis resulted in greater acidosis in preconditioned than in control hearts. For these reasons, the faster initial recovery from acidosis in preconditioned hearts may be a consequence of the lower starting pH_i_. Indeed, de Albuquerque et al. (1995) recently showed that, in a similar condition, the rate of pH_i_ recovery is similar in control and preconditioned rat hearts. More recently, it has been demonstrated that NHE activity did not contribute to the cardioprotection mediated by ischemic preconditioning. Indeed, inhibition of Na^+^/H^+^ exchanger isoform-1 (NHE1) is protective in adult myocardium in a model of ischemia followed by reperfusion; however, the effect is unclear in immature myocardium (Cun et al., 2007). More important, the administration of HOE, a NHE inhibitor, did not prevent the protection induced by preconditioning, thus suggesting that NHE inhibition during the prolonged ischemic period may enhance the protection afforded by preconditioning (Shipolini et al., 1997). Accordingly, another group demonstrated that both NHE inhibition and ischemic preconditioning eliminated the increase in intramyocyte Na^+^ content that otherwise occurred with cardioplegic arrest and reperfusion in a porcine model. Because their mechanisms are distinct, the authors proposed that an additive beneficial effect against ischemia-reperfusion injury can be achieved by using NHE inhibition together with a preconditioning stimulus as prereperfusion therapy (Goldberg et al., 2002).

### NHE and NKCC role during ischemic post-conditioning

Up until now, there is few information about the role of NHE1 exchanger in brain post-conditioning since many studies have demonstrated its neurodegenerative role in brain ischemia upon activation. According to these data, the ischemic neuroprotection induced by post-conditioning may be associated with the downregulation of NHE1 expression or activity. These hypothesis are supported by few experimental evidences. In this regard, some information were obtained in the heart where it has been demonstrated the role of NHE in a delayed recovery of pH_i_ after heart post-conditioning. Indeed, it has been suggested that prolongation of acidosis during reperfusion is important for the post-conditioning-mediated neuroprotection. Inserte et al. demonstrated that such post-conditioning protocols that significantly prolong pH_i_ recovery during initial reperfusion are protective for the heart. Accordingly, there is a narrow inverse correlation between the level of the delay and the extent of cell death, demonstrating the key role of the delay of intracellular acidosis in the protection mediated by post-conditioning (Inserte et al., 2009). The normalization of pH_i_ during reperfusion is mediated by the combined action of different transport systems, including Na^+^/H^+^-exchanger, Na^+^/HCO3^−^ symport, and H^+^-coupled lactate efflux (Vandenberg et al., 1993). These systems appear to be mainly redundant, since inhibition of one of them does not result in significant delay in pH_i_ recovery. For instance, inhibition of Na^+^/H^+^-exchanger by selective agents administered at the onset of reperfusion has a very small effect on pH_i_ recovery (Docherty et al., 1997; Ten Hove and Van Echteld, 2004). By contrast, simultaneous inhibition of Na^+^/H^+^-exchanger and Na^+^/HCO3^−^ symport delay normalization of pH_i_ (Docherty et al., 1997; Schäfer et al., 2000; Inserte et al., 2008).

The concept that pH_i_ correction was correlated to the washout of lactate, H^+^ and CO_2_ was supported by the strong correlation between the delay in pH_i_ recovery and the levels of lactate measured in the coronary effluent (blood outflow through coronaries) during the first 2 min of reperfusion (Vandenberg et al., 1993; Inserte et al., 2008). Thus, reduced catabolite washout caused an attenuation of the transmembrane H^+^ gradient and a decreased activity of Na^+^/H^+^-exchanger and Na^+^/HCO3^−^ symport.

The protective effect of prolongation of intracellular acidosis during reperfusion has been solidly demonstrated in different models, including isolated cardiomyocytes (Schäfer et al., 2000) and intact hearts (Kitakaze et al., 1988; Ohashi et al., 1996; Preckel et al., 1998; Inserte et al., 2008). Intriguingly, there is a correlation between delayed acidosis and other cardioprotective pathways during post-conditioning such as Reperfusion Injury Salvage Kinases (RISK) (Fujita et al., 2007). According to this theory, alkalotic buffer reduced Akt and ERK phosporylation in post-conditioned myocardium.

Regarding the role of Na^+^-dependent chloride transporter (NKCC) during brain post-conditioning few information are available. In a recent work it has been demonstrated that in hippocampal neurons exposed to hypoxia conditioning mimiking an *in vitro* post-conditioning, NKCC1 is strongly activated through binding with STE20/SPS1-related proline/alanine-rich kinase (SPAK) (Yang et al., 2013). This finding could be considered as starting point for future studies.

### Role of acid-sensing cation channels (ASIC) in brain conditioning

Acid-Sensing Ion Channels (ASICs) are neuronal voltage-insensitive cationic channels activated by extracellular protons. ASIC proteins are a subfamily of the ENaC/Deg superfamily of ion channels. ASICs are potential drug targets for treating a wide variety of conditions linked to both the CNS and PNS. Acid-sensing cation channels (ASIC) have been extensively studied both in physiological and in pathological conditions for their wide distribution in the nervous system and since they represent key targets in many different aspects of acidosis, a condition that occurs during ischemic stroke, as previously analyzed (Waldmann et al., 1997; Wemmie et al., 2006). Until now, six ASIC subunit proteins, encoded by four genes, have been identified: ASIC1a, ASIC1b, ASIC2a, ASIC2b, ASIC3, and ASIC4; among these, the subtypes 1a, 2a, and 2b are expressed in neurons of the CNS. All ASICs belong to the degenerin/epithelial Na^+^ channel (DEG/ENaC) superfamily, which are Na^+^- selective cation channels sensitive to amiloride (Crowell and Kaufmann, 1961; Sutherland et al., 2000). Blockade of ASIC1a and deletion of the ASIC1 gene rescued neurons from ischemic cell death. In CNS neurons, ASIC1a responds to extracellular pH reduction ranging from 6.9 to 5.0 to generate rapid depolarizing currents (Waldmann et al., 1997), and its activation enhances the probability of action potential initiation (Vukicevic and Kellenberger, 2004). ASIC1a channels are activated at the pH values associated with CNS diseases (ischemia, ~pH 6.5–6.0; seizure, ~pH 6.8; AD, ~pH 6.6). In the case of homomeric ASIC1a channels, acid activation also induces Ca2^+^ entry directly through these channels (Crowell and Kaufmann, 1961; Nedergaard et al., 1991; Dietrich and Morad, 2010). In addition, the ASIC-mediated membrane depolarization may facilitate the activation of voltage gated Ca^2+^ channels and NMDA receptor-gated channels (Gao et al., 2005), further promoting neuronal excitation and [Ca^2+^]_i_ accumulation.

Acidosis usually occurs during many central nervous system (CNS) diseases. Indeed, ischemic brain pH falls to 6.0 due to the accumulation of lactic acid and to the protons produced by ATP hydrolysis (Nedergaard et al., 1991; Gao et al., 2005). So the reasonable strategy to contrast pH lowering, sustained by ASICs activation, is to block its activity, since acidosis is a common feature of acute neurological conditions such as ischemic stroke, brain trauma, and epileptic seizures (Rehncrona, 1985). It is rational to suggest that restoration of acid–base balance and blockade of the down-stream pathways of acidosis may represent two promising strategies to avoid the consequences of acidosis.

In this direction ASIC involvement in neuroprotection elicited in the brain by pre- and post-conditioning has been investigated (Pignataro et al., 2007).

Indeed, Pignataro and colleagues demonstrated that ASIC1a is downregulated during both preconditioning and post-conditioning and that this downregulation was dependent on p-Akt. Particularly, p-Akt is up-regulated during ischemic pre- and post-conditioning. In fact, during harmful ischemia, Akt is transiently phosphorylated, and then activated for a short time interval after reperfusion (Beg et al., 2008), whereas after brain conditioning the phosphorylation of Akt persists longer, even 24 h later, the same time interval at which an ASIC1a downregulation occurs. Interestingly, selective p-Akt inhibition prevented ASIC1a downregulation thus reverting the conditioning-induced neuroprotection.

That p-Akt is the mediator of the downregulation of ASIC1a during pre- and post-conditioning are supported by previous papers showing that Reperfusion Injury Salvage Kinasesì (RISKs), including Akt (Nedergaard et al., 1991; Xu and Xiong, 2007; Beg et al., 2008; Pignataro et al., 2011a) are activated during conditioning. In conclusion, since reduction of ASIC1a is a key factor in the neuroprotective effect of pre- and post-conditioning, the downregulation of its expression and activity might be a potential strategy to ameliorate the ischemic damage. Besides ASIC1a, ASIC2a has also been linked to ischemic preconditioning neuroprotection. In fact, the upregulation of ASIC2a is associated to the neuroprotective effect of ischemic preconditioning on global brain ischemia (Miao et al., 2010).

### Role of sodium/calcium exchanger (NCX) during brain conditioning

The sodium-calcium exchanger (Na^+^/Ca^2+^ exchanger, NCX, or exchange protein) is an antiporter membrane protein that removes calcium from cells. It consists of nine transmembrane segments that can mediate Ca^2+^ and Na^+^ fluxes across the plasma membrane. Depending on the intracellular concentrations of Ca^2+^, [Ca^2+^]_i_, and Na^+^, [Na^+^]_i_, NCX can operate either in the forward mode, coupling the uphill extrusion of Ca^2+^ to the influx of Na^+^ ions, or in the reverse mode, mediating the extrusion of Na^+^ and the influx of the Ca^2+^ ions (Pignataro et al., 2004). The NCX is considered one of the most important cellular mechanisms for removing Ca2^+^. The maintenance of Na^+^ and Ca^2+^ homeostasis, mediated by this exchanger, is a crucial physiological phenomenon in neuronal and non-neuronal cells. NCX countertransports four or three Na^+^ ions in change of one Ca^2+^ ion (Reeves and Hale, 1984; Fujioka et al., 2000; Kang and Hilgemann, 2004); it is a high-capacity and low-affinity ionic transporter. When intracellular Ca^2+^ concentrations ([Ca^2+^]_i_) increase this exchanger mediates the entrance of sodium ions and the extrusion of calcium ions in tight dependence of their electrochemical gradient. The way in which the antiporter works (forward or reverse) depends on several factors such as membrane potential, Na^+^ and Ca^2+^ gradient. Therefore, NCX represents an important actor in the regulation of pathophysiological and physiological responses to increases of [Ca^2+^]_i_ and [Na^+^]_i_ (Haddad and Jiang, 1997; Dirnagl et al., 1999; Annunziato et al., 2004) In particular, three distinct NCX genes, ncx1-3, and several splicing variants have been so far described (Nicoll et al., 1990, 1996). The CNS is the only organ that expresses all three NCX gene products. NCX is particularly expressed in neurons at the level of synapses (Canitano et al., 2002) where, in the course of an action potential, Ca^2+^ crosses the plasma membrane. Calcium entry allows the fusion of synaptic vesicles with the plasmamembrane and permits the neurotransmitter release. After that, plasma membrane Ca^2+^ ATPase and NCX mediate the extrusion of the residual intracellular Ca^2+^. In presence of [Ca^2+^]_i_ higher than 500 nM, NCX become the prevalent Ca^2+^ extrusion mechanism (Pignataro et al., 2014). At the same time, the deregulation of [Ca^2+^]_i_ and [Na^+^]_i_ homeostasis may mediate the damage of neuronal and glial cells occurring after stroke and other neurodegenerative disorders.

### Na^+^/Ca^2+^ exchanger in brain preconditioning

It is well-known that NCX may play a role in the maintenance of ionic homeostasis during ischemic conditioning (Pignataro et al., 2004, 2012; Molinaro et al., 2013). In this respect, NCX is downregulated during brain ischemia and this downregulation occurs in a manner dependent on the exchanger isoform and on the brain region interested by the damage (Pignataro et al., 2004; Boscia et al., 2006). It has been recently demonstrated that NCX1 and NCX3 isoforms represent two new effectors of the preconditioning-mediated neuroprotection (Pignataro et al., 2012). In fact, while the absence of NCX1 and NCX3 is able to partially prevent the neuroprotection mediated by ischemic preconditioning, the pro-survival factor p-Akt mediated preconditioning-induced brain protection by inducing an upregulation of NCX1 and NCX3. In this field, these results have been obtained in an experimental model of ischemic preconditioning in which preconditioning was mediated in rats by 30 min occlusion of the middle cerebral artery (MCA), followed by a subsequent harmful stimulus of 100 min of MCA occlusion, applied 72 h after the preconditioning stimulus (Pignataro et al., 2012). The correlation between NCX and p-Akt was demonstrated also by confocal fluorescence experiments, that showed a colocalization among NCX1, NCX3, and p-Akt in the temporoparietal cortex of preconditioned rats (Formisano et al., 2008; Pignataro et al., 2012). Moreover, the PI3-K inhibitor, LY294002, reverts the protective outcome elicited by ischemic preconditioning, thus giving further support to the role of p-Akt in this phenomenon (Endo et al., 2006; Pignataro et al., 2008). The overexpression p-Akt-dependent of NCX1 and NCX3 elicited by brain preconditioning may be considered a response of cells to overcome the deregulation of ionic homeostasis occurring in the brain under anoxic conditions. On the other hand, p-Akt is not the only transducer able to activate NCX1 and NCX3. Indeed, Valsecchi and colleagues underlineed that after ischemic preconditioning, hypoxia-inducible factor, HIF-1α, is strongly increased and mediated the increased expression of NCX1 expression, thus contributing to brain protection induced by ischemic preconditioning (Valsecchi et al., 2011) and suggesting that ncx1 gene represents a new HIF-1 target (Valsecchi et al., 2011). Summarizing, NCX1 and NCX3 overexpression may be considered as a mechanism that preconditioning activates in neurons and glial cellsin order to overcome the loss of ionic homeostasis caused by harmful ischemia. Interestingly, the activation of these mechanisms are long lasting, since NCX1 and NCX3 upregulation is still present 72 h after preconditioning induction, thus suggesting that these two isoforms can be included in the list of effectors able to mediate the so-called delayed preconditioning (Pignataro et al., 2013a).

### Na^+^/Ca^2+^ exchanger in brain post-conditioning

Regarding NCX role during brain post-conditioning, NCX3 has been confirmed as a molecular effector involved in the neuroprotection (Pignataro et al., 2011b). In particular, interesting results have been obtained in an experimental model of ischemic post-conditioning, performed in the laboratory of Pignataro and colleagues by subjecting adult male rats to 10 min of MCAO 10 min after 100 min of MCAO. Results showed that p-Akt expression after post-conditioning increased and timely mirrors the increase in NCX3 expression. On the other hand, NCX3 silencing reverted the post-conditioning-mediated neuroprotection and the selective inhibition of p-Akt prevented NCX3 upregulation and the neuroprotection (Pignataro et al., 2011b). NCX3 overexpression during post-conditioning may contribute to counteract the ionic homeostasis dysregulation. By contrast NCX1 silencing did not revert the post-conditioning-induced neuroprotection, thus showing that NCX1 does not play a relevant role during post-conditioning. This result can be explained by the observation that unlike NCX1 and NCX2, the isoform NCX3 may be still operative during ATP depletion (Linck et al., 1998; Secondo et al., 2007). In addition, the differences in NCX1 and NCX3 promoters render NCX3 a better target for the prosurvival kinase cAMP response element-binding protein (CREB), an Akt downstream player (Gabellini et al., 2003). After post-conditioning, the phosphorylation of Akt is present longer (Pignataro et al., 2008) and timely parallels the intervals of NCX3 upregulation. Furthermore, confocal experiments in the temporoparietal confirmed that the increased expression of these two proteins after post-conditioning occurs in the same cells. These considerations, beside showing that p-Akt palys a key role in neuroprotection, more important suggest NCX3 as one of the additional p-Akt effectors involved in the neuroprotective effect of ischemic post-conditioning.

### Role of plasma membrane Ca^2+^ ATPase (PMCA) in brain conditioning

The plasma membrane Ca2^+^ ATPase (PMCA) is a transport protein in the plasma membrane of cells that functions removing calcium (Ca2^+^) from the cell; so it is also an important regulator of the calcium concentration in the extracellular space. This protein is the only plasmamembrane high affinity Ca^2+^ transporting system. It removes Ca^2+^ from the cytosol in all eukaryotic cells, and for most of them it is the only Ca^2+^ exporting system together with Na^+^/Ca^2+^ exchanger which represent the principal Ca^2+^ exchanger in heart cells and neurons (Jensen et al., 1993). The high Ca^2+^ affinity of the pump, however renders it the only system capable of fine-tuning its exchanges with the external ambient (Brini et al., 2013).

Conversely to other plasma membrane buffering systems, such as NCX and ATPases, plasma membrane Ca^2+^ -ATPase may export intracellular Ca^2+^ even with relatively lower transport capacity during prolonged membrane hyperpolarization and during conditions of increased intracellular Na^+^ concentrations, as occurred under ischemic conditions. Ohta et al. (1996) suggested that ischemia-tolerant CA1 neurons, in a model of bilateral common carotid arteries occlusion in gerbils, export more efficiently a larger amount of Ca^2+^ (Kato et al., 2005).

Moreover, in gerbils, ischemic preconditioning by pre-exposure to 2-min ischemia is extremely effective in protecting against ischemic cell death since there is a modification of the electrophysiological properties of CA1 neurons (Kawai et al., 1998; Shimazaki et al., 2000). In this experimental conditions, expression of PMCA1 is increased in the hippocampus following 2-min ischemia (Kato et al., 2005). The same authors evaluated PMCA1 expression also in a model of tolerance induced by a low dose treatment with 3-Nitro Propionic Acid (3-NPA). They observed an increase in the expression of Bcl-2 in the hippocampus in both the models of preconditioning. However, PMCA1 increased just in the 2-min ischemia model and not in the 3-NPA model, thus suggesting that it plays an important but not essential role in the enhancement of ischemic tolerance (Kato et al., 2005).

## Conclusions

The identification of the players involved in the control of ionic homeostasis during pre and post-conditioning should provide more direct opportunities for translational neuroprotection trials. Indeed, the present review, summarizing the specific effect of the plasmamembrane proteins involved in the maintenance of cellular ionic concentration, would provide information of which transporters needed to be activated or inhibited in order to protect the brain by the ischemic injury.

### Conflict of interest statement

The authors declare that the research was conducted in the absence of any commercial or financial relationships that could be construed as a potential conflict of interest.
